# Supplementary material for: Foliar Application of Different Iron Sources Improves Morpho-Physiological Traits and Nutritional Quality of Broad Bean Grown in Sandy Soil
Source: Plants (Basel). 2022 Oct 2;11(19):2599. doi: 10.3390/plants11192599 (PMC9572197; doi:10.3390/plants11192599)
Supplement: Supplementary file 1 [file plants-11-02599-s001.zip › plants-1922091-supplementary.pdf]

Supplementary Data

Supplementary table S1 (S1). Pearson's correlation analysis between the tested variables of fava bean grown in sandy soil and treated with different sources of iron fertilizers

Variables	Plant height	Leaf area	No. of branches	Shoot F.W	Shoot D.W	No. of seed	Biological yield	No.of pods	Total Chl	Carot.	*Pn*	*SC*	WUE	Seed N	Seed Fe	Seed Zn	Total Carbo.	Protein	IAA	GA3	ABA
Plant height	**1**																				
Leaf area	**0.75**	**1**																			
No. of branches	**0.90**	0.22	**1**																		
Shoot F.W	**0.92**	**0.93**	**0.59**	**1**																	
Shoot D. W	**0.92**	**0.93**	**0.68**	**0.77**	**1**																
No. of seed	0.18	**0.60**	**0.91**	**0.67**	0.25	**1**															
Biological yield	**0.92**	**0.91**	**0.96**	**0.78**	**0.74**	**0.88**	**1**														
No.of pods	**0.88**	**0.79**	**0.87**	**0.82**	**0.50**	**0.95**	**0.86**	**1**													
Total Chl.	**0.89**	**0.95**	**0.95**	**0.90**	**0.92**	**0.96**	**0.94**	**0.80**	**1**												
Carot.	**0.91**	**0.86**	0.41	**0.87**	**0.66**	**0.88**	**0.92**	**0.88**	**0.84**	**1**											
Pn	**0.93**	**0.92**	**0.98**	**0.98**	**0.83**	**0.94**	**0.91**	**0.91**	**0.91**	**0.79**	**1**										
SC	**0.96**	**0.68**	**0.98**	**0.74**	**0.89**	**0.86**	**0.92**	**0.76**	**0.81**	**0.64**	**0.71**	**1**									
WUE	**0.91**	0.17	**0.97**	**0.82**	**0.61**	**0.77**	**0.97**	**0.84**	**0.79**	0.20	**0.98**	**0.83**	**1**								
Seed N	**0.87**	**0.94**	**0.95**	**0.94**	**0.74**	**0.98**	**0.90**	**0.84**	**0.89**	**0.81**	**0.79**	**0.94**	**0.86**	**1**							
Seed Fe	**0.52**	**0.88**	**0.56**	**0.74**	**0.85**	**0.90**	**0.97**	**0.90**	**0.68**	**0.77**	**0.82**	**0.82**	**0.88**	**0.98**	**1**						
Seed Zn	**0.64**	**0.78**	**0.99**	**0.72**	**0.61**	**0.86**	**0.75**	**0.88**	**0.82**	**0.96**	**0.53**	**0.56**	**0.77**	**0.77**	**0.79**	**1**					
Total Carb.	**0.90**	**0.77**	**0.74**	**0.83**	**0.78**	**0.85**	**0.97**	**0.88**	**0.97**	**0.92**	**0.87**	**0.98**	**0.83**	**0.55**	**0.78**	**0.73**	**1**				
Protein	**0.95**	**0.86**	0.25	**0.80**	**0.81**	**0.91**	**0.97**	**0.76**	**0.70**	**0.91**	0.46	**0.79**	**0.84**	**0.88**	**0.51**	**0.65**	**0.85**	**1**			
IAA	**0.93**	**0.66**	**0.92**	**0.88**	**0.55**	**0.74**	**0.92**	**0.83**	**0.59**	**0.90**	**0.85**	**0.88**	**0.78**	**0.77**	**0.88**	**0.54**	**0.75**	**0.81**	**1**		
GA3	0.20	**0.59**	**0.90**	0.24	**0.83**	**0.88**	**0.93**	**0.91**	0.42	**0.84**	**0.87**	**0.83**	**0.70**	**0.66**	**0.88**	**0.78**	**0.79**	**0.58**	**0.88**	**1**	
ABA	**-0.58**	**-0.63**	**-0.57**	**-0.73**	**-0.62**	-0.38	**-0.84**	**-0.75**	**-0.56**	**-0.57**	**-0.55**	**-0.62**	**-0.57**	-0.47	**-0.50**	-0.42	**-0.58**	**-0.53**	**-0.40**	**-0.67**	**1**

Values in bold are different from 0 with a significance level alpha=0.05. F.W= fresh weight, D.W. = dry weight, Chl. = chlorophyll, Carot. = carotenoids, *Pn*= photosynthesis rate, WUE= water use efficiency, *SC*= stomatal conductance, Carbo. = Carbohydrate.

(Table S1 in word format) Supplementary Data

Supplementary table S1 (S1). Pearson’s correlation analysis between the tested variables of fava bean grown in sandy soil and treated with different sources of iron fertilizers

Variables	Plant height	Leaf area	No. of branches	Shoot F.W	Shoot D.W	No. of seed	Biological yield	No.of pods	Total Chl	Carot.	*Pn*	*SC*	WUE	Seed N	Seed Fe	Seed Zn	Total Carbo.	Protein	IAA	GA3	ABA
Plant height	**1**																				
Leaf area	**0.75**	**1**																			
No. of branches	**0.90**	0.22	**1**																		
Shoot F.W	**0.92**	**0.93**	**0.59**	**1**																	
Shoot D. W	**0.92**	**0.93**	**0.68**	**0.77**	**1**																
No. of seed	0.18	**0.60**	**0.91**	**0.67**	0.25	**1**															
Biological yield	**0.92**	**0.91**	**0.96**	**0.78**	**0.74**	**0.88**	**1**														
No.of pods	**0.88**	**0.79**	**0.87**	**0.82**	**0.50**	**0.95**	**0.86**	**1**													
Total Chl.	**0.89**	**0.95**	**0.95**	**0.90**	**0.92**	**0.96**	**0.94**	**0.80**	**1**												
Carot.	**0.91**	**0.86**	0.41	**0.87**	**0.66**	**0.88**	**0.92**	**0.88**	**0.84**	**1**											
Pn	**0.93**	**0.92**	**0.98**	**0.98**	**0.83**	**0.94**	**0.91**	**0.91**	**0.91**	**0.79**	**1**										
SC	**0.96**	**0.68**	**0.98**	**0.74**	**0.89**	**0.86**	**0.92**	**0.76**	**0.81**	**0.64**	**0.71**	**1**									
WUE	**0.91**	0.17	**0.97**	**0.82**	**0.61**	**0.77**	**0.97**	**0.84**	**0.79**	0.20	**0.98**	**0.83**	**1**								
Seed N	**0.87**	**0.94**	**0.95**	**0.94**	**0.74**	**0.98**	**0.90**	**0.84**	**0.89**	**0.81**	**0.79**	**0.94**	**0.86**	**1**							
Seed Fe	**0.52**	**0.88**	**0.56**	**0.74**	**0.85**	**0.90**	**0.97**	**0.90**	**0.68**	**0.77**	**0.82**	**0.82**	**0.88**	**0.98**	**1**						
Seed Zn	**0.64**	**0.78**	**0.99**	**0.72**	**0.61**	**0.86**	**0.75**	**0.88**	**0.82**	**0.96**	**0.53**	**0.56**	**0.77**	**0.77**	**0.79**	**1**					
Total Carb.	**0.90**	**0.77**	**0.74**	**0.83**	**0.78**	**0.85**	**0.97**	**0.88**	**0.97**	**0.92**	**0.87**	**0.98**	**0.83**	**0.55**	**0.78**	**0.73**	**1**				
Protein	**0.95**	**0.86**	0.25	**0.80**	**0.81**	**0.91**	**0.97**	**0.76**	**0.70**	**0.91**	0.46	**0.79**	**0.84**	**0.88**	**0.51**	**0.65**	**0.85**	**1**			
IAA	**0.93**	**0.66**	**0.92**	**0.88**	**0.55**	**0.74**	**0.92**	**0.83**	**0.59**	**0.90**	**0.85**	**0.88**	**0.78**	**0.77**	**0.88**	**0.54**	**0.75**	**0.81**	**1**		
GA3	0.20	**0.59**	**0.90**	0.24	**0.83**	**0.88**	**0.93**	**0.91**	0.42	**0.84**	**0.87**	**0.83**	**0.70**	**0.66**	**0.88**	**0.78**	**0.79**	**0.58**	**0.88**	**1**	
ABA	**-0.58**	**-0.63**	**-0.57**	**-0.73**	**-0.62**	-0.38	**-0.84**	**-0.75**	**-0.56**	**-0.57**	**-0.55**	**-0.62**	**-0.57**	-0.47	**-0.50**	-0.42	**-0.58**	**-0.53**	**-0.40**	**-0.67**	**1**

Values in bold are different from 0 with a significance level alpha=0.05. F.W= fresh weight, D.W. = dry weight, Chl. = chlorophyll, Carot. = carotenoids, *Pn*= photosynthesis rate, WUE= water use efficiency, *SC*= stomatal conductance, Carbo. = Carbohydrate.
